# Harnessing DNA Nanotechnology and Chemistry for Applications in Photonics and Electronics

**DOI:** 10.1021/acs.bioconjchem.2c00286

**Published:** 2022-09-19

**Authors:** Katherine E. Dunn, Alistair Elfick

**Affiliations:** School of Engineering, Institute for Bioengineering, University of Edinburgh, The King’s Buildings, Edinburgh, EH9 3DW, Scotland, U.K.

## Abstract

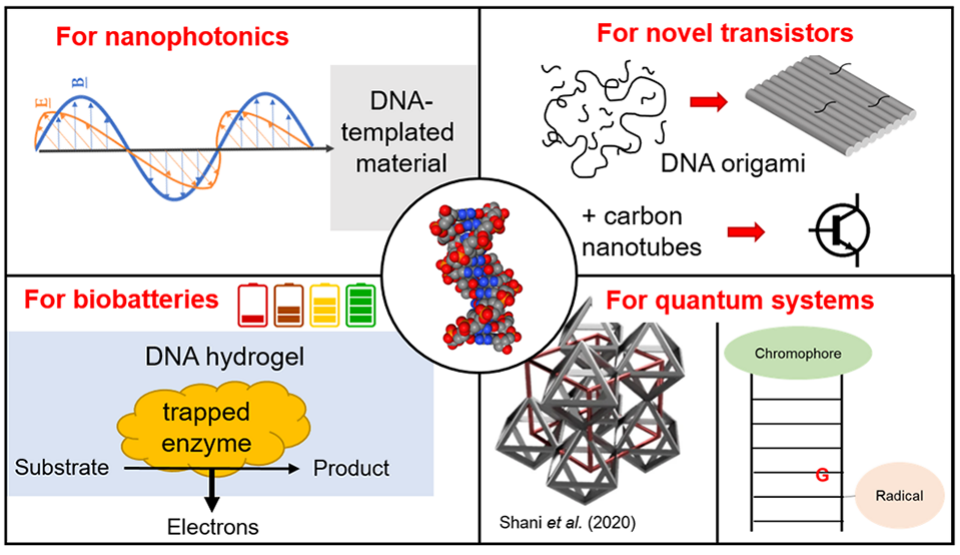

Many photonic and electronic devices rely on nanotechnology and nanofabrication, but DNA-based approaches have yet to make a significant commercial impact in these fields even though DNA molecules are now well-established as versatile building blocks for nanostructures. As we describe here, DNA molecules can be chemically modified with a wide variety of functional groups enabling nanocargoes to be attached at precisely determined locations. DNA nanostructures can also be used as templates for the growth of inorganic structures. Together, these factors enable the use of DNA nanotechnology for the construction of many novel devices and systems. In this topical review, we discuss four case studies of potential applications in photonics and electronics: carbon nanotube transistors, devices for quantum computing, artificial electromagnetic materials, and enzymatic fuel cells. We conclude by speculating about the barriers to the exploitation of these technologies in real-world settings.

## Introduction

Since the field of DNA nanotechnology was founded by the late Ned Seeman,^1^ researchers have succeeded in building an enormous variety of self-assembling nanostructures using DNA molecules as building blocks,^2,3^ often using the technique of DNA origami as invented by Paul Rothemund.^4^ Through the specificity of base pairing, DNA nanotechnology offers unsurpassed programmability in achieving exceptionally accurate self-assembly in 3D, and evaluation of patent filings and creation of companies suggests that the field is now sufficiently mature to support commercialization.^5^ Many proposed applications lie in biomedicine,^6^ but there are also valuable opportunities in physics and engineering that have so far been underexploited. Examples include improved manufacture of nanoscale devices for electronics and computing,^7^ construction of photonic devices that provide new ways to manipulate light,^8^ and the generation of electricity. For these purposes the key advantages of DNA are the ability to chemically modify DNA for tethering to surfaces or cargoes, the possibility of using DNA structures for spatially organizing moieties with nanoscale precision, and the potential for using DNA to build nanoscale templates. The benign conditions for assembly (aqueous solution, no extreme chemicals or temperatures) bring “green” credentials as an added bonus. Here, we discuss these attributes and present case studies demonstrating the use of DNA nanotechnology to enable advances in photonics, electronics, computing, and electricity generation.

### Chemical Modifications

**Figure 1:**
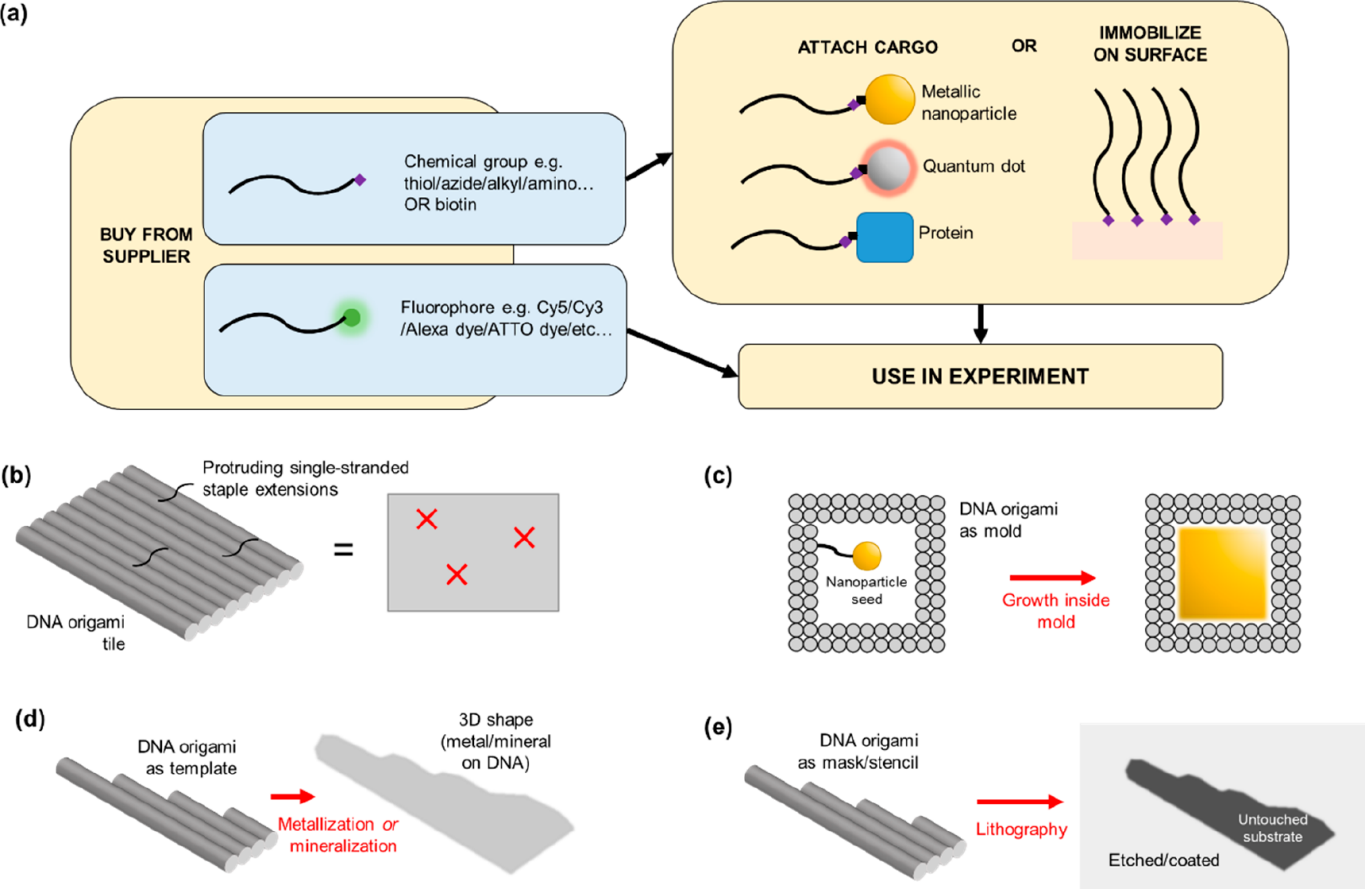
(a) Selection of some of the key chemical modifications and cargoes that can be used with DNA oligonucleotides. Some modifications can be acquired with ease from commercial suppliers, whereas more complex conjugations require extended laboratory protocols to be carried out by the end user. The black curved line represents a DNA oligonucleotide, the shapes represent modifications/cargoes as shown by the labels, and the black square represents a linker moiety. (b) Use of DNA origami as a nanoscale breadboard. Individual staples are extended such that single-stranded DNA segments protrude from the surface of the origami tile. As each staple has a unique sequence, corresponding to a precise location in the structure, the position of the extensions is determined to a high degree of precision and this may be used for spatial organization of functional groups or bioconjugates. (c) Casting: Use of a DNA origami shell as a mold for the growth of metallic structures around a nanoparticle seed. (d) Metallization/mineralization: DNA nanostructure of the desired shape is coated in a substance such as metal, silica etc., resulting in an object having approximately the shape of the original nanostructure. (e) Lithography: Different variations on the process exist. In the approach shown here, the DNA nanostructure is used to construct a mask, protecting an underlying substrate (dark gray) from a coating/etching (pale gray).

DNA synthesis companies offer a rich catalogue of chemical modifications of DNA, on both the backbone and the bases. Chemical modifications suitable for tethering include biotin, thiol, amino, alkyne, or azide groups.^9^ Such modifications are commonly used to immobilize DNA constructs, as in the use of thiol–gold bonds to form a surface-bound DNA nanostructure monolayer.^10^ In a more exotic example, DNA oligonucleotides modified with alkyne (octadiynyl) or azide groups were used, in combination with copper-catalyzed azide–alkyne click chemistry, to selectively coat highly doped silicon-based ring resonators that had been functionalized with the appropriate complementary group.^11^ Chemical modification is also key for attaching functional cargoes to DNA nanostructures, including fluorophores,^12^ quantum dots,^13^ other nanoparticles,^14^ proteins,^15^ etc. (Figure 1a).

### Precise Spatial Localization

Many applications of DNA nanotechnology depend on the fact that each constituent oligonucleotide in a DNA nanostructure is unique and may be tagged independently with a specific cargo, enabling the cargoes to be placed at precise positions in the final structure (Figure 1b). Examples of cargoes include proteins such as enzymes.^16,17^ Recently a number of studies have shown that spatial control over DNA nanostructure cargoes can be used to form the specific patterns of biological signaling molecules that are required to cause cells to undergo apoptosis^18,19^ or induce immune activation.^20^ Such work demonstrates in a biological setting the capability of DNA nanostructures to arrange cargoes precisely, which is also extremely valuable for applications in electronics, nanophotonics, and other engineering-based technologies. One such example is the use of a DNA origami breadboard for construction of a nanoparticle heterotrimer, where energy transfer between two gold nanoparticles was mediated by a silver nanoparticle placed in the gap between them.^21^ Further examples will be discussed below.

### Templating for Nanofabrication

Many conventional electronic and photonic technologies rely on nanofabrication. Existing nanofabrication manufacturing approaches can be classed as top down or bottom up.^22^ Top-down approaches such as photolithography, electron beam lithography, scanning probe lithography, molecular beam epitaxy, liquid phase epitaxy, and focused ion beam lithography can produce sub-100 nm geometries with features smaller than 20 nm; however, they are fundamentally hamstrung by their inability to deliver such features over centimeter-scale surfaces or out-of-plane, with affordability and speed. In contrast, DNA nanotechnology offers an alternative route for nanofabrication, via a versatile combination of customizable nanoscale shapes and chemical reactions that enable them to act as three-dimensional templates^23^ for metallization, mineralization, lithography, and casting (Figure 1c–e). DNA nanostructures can be used for many applications other than the direct assembly of inorganic structures, for example as a 3D mask for reactive ion etching,^24^ as a template for assembly of stamps for soft lithography,^25^ or as a means to deliver site-specific doping of semiconductor substrates.^26^ Recent studies have also begun to shed more light on the underlying mechanisms of processes that involve depositing material on the DNA nanostructure.^27,28^

## Case Studies

### Computing and Carbon Nanotube Transistors

Semiconductor devices and systems underpin modern electronics. The state of the art in the semiconductor industry is reviewed annually by a team sponsored by IEEE (Institute of Electrical and Electronics Engineers), resulting in an international road map. The most recent such road map^29^ shows that the semiconductor industry continues to try to squeeze more and more computing power into the same space without overheating. This involves moving to smaller feature sizes, often exploiting extreme ultraviolet lithography (at great expense and complexity), but also utilizing the third dimension, while changing both hardware and software to reduce power consumption.

Conventional computing is based on bits, which can be in one of two discrete states. Information processing is normally carried out by transistors, which often act like sophisticated electronic switches. Modern electronic systems are usually underpinned by silicon-based technology, but alternative approaches are being investigated, including devices based on carbon nanotubes.^30^ Here, the carbon nanotube (CNT) acts as an electron channel between source and drain electrodes. The current is switched on or off by means of a gate electrode. A variety of CNT transistors have been tested, but fabrication and performance challenges remain. DNA nanotechnology could provide a valuable tool for the construction of devices of this type.

**Figure 2:**
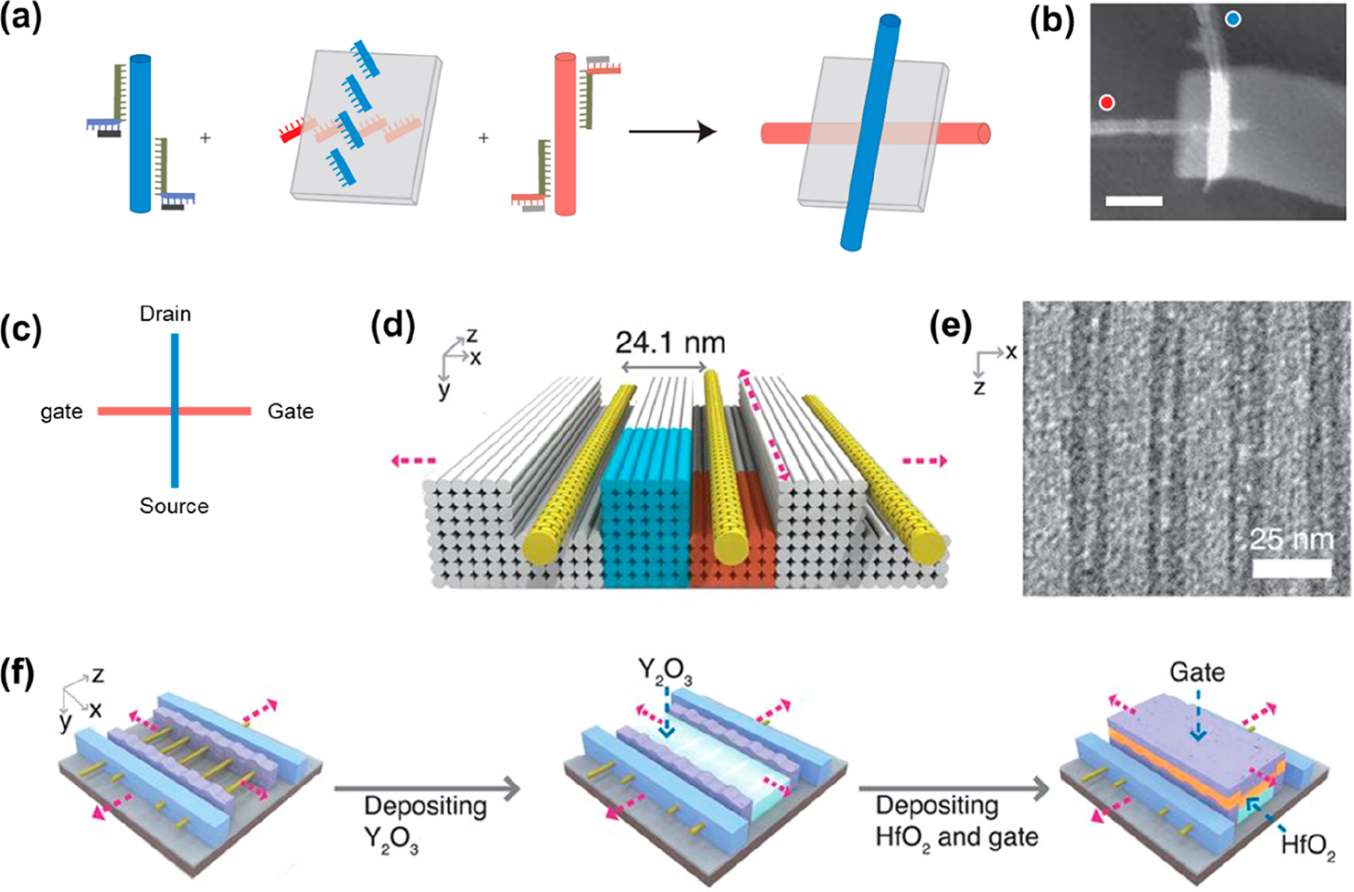
(a) Method for attaching carbon nanotubes site specifically in a cross shape to a DNA origami substrate. (b) Image acquired using atomic force microscopy, showing correct assembly. The scale bar is 50 nm. The two nanotubes (long thin structures) have different types of DNA tags (indicated by red and blue labels) for attachment to a rectangular DNA origami tile, which itself is connected to a DNA ribbon that extends at an angle toward the bottom right of the image. (c) Sketch showing how the nanotubes are connected to electrodes in a transistor-like configuration. (d) Arranging carbon nanotubes in an array with precisely determined intertube separation. (e) Transmission electron micrograph of the structure from (d). (f) Carbon nanotube field-effect transistors fabricated using the method of (d) and (e). In the leftmost image, the purple objects are the source and drain electrodes, carbon nanotubes are shown in yellow, and the blue blocks are metal bars. (a and b) Reprinted with permission from ref (31). Copyright 2009 Springer Nature. (d and e) Reprinted with permission from ref (32). Copyright 2020 AAAS. (f) Reprinted with permission from ref (33). Copyright 2020 AAAS.

In 2010, a rectangular DNA origami tile was used to guide assembly of CNTs^31^ (Figure 2a,b). Two CNTs were attached to the tile in a cross-like formation, and electrodes were fabricated for electronic characterization (Figure 2c). Of six devices, one exhibited transistor-like behavior. Subsequently, it was shown that an array of parallel CNTs could be formed with a similar technique.^32^ In this case the origami substrate was a three-dimensional block that contained multiple trenches, and the spacing of the trenches enabled control over the separation of the CNTs (Figure 2d,e). It was demonstrated that this strategy could be used to build CNT field-effect transistors^33^ (Figure 2f).

Via templated metallization (see above), DNA nanostructures can also enable fabrication of interconnects, wiring that connects different devices in a circuit. It has been shown that conducting metal–semiconductor junctions can be templated by DNA origami^34^ and complex branched metal nanostructures can be made using DNA origami molds.^35^ Organic materials can also be used, and individual polymers can be routed in curved patterns on the surface of DNA origami tiles.^36^ Such polymers could potentially be made conducting for use in technologies that would benefit from flexible electronic circuitry, such as wearable health monitoring devices or bendable smartphones.

Future studies could use a combination of the technologies described here to produce integrated circuits with multiple components and complex wiring pathways. For a broader review of DNA-based nanoelectronics, the reader is referred to the review by Hui et al.^7^

### Quantum Computing

One trend identified in the International Roadmap for Devices and Systems is the development of quantum computing,^29^ which is based on qubits.^37^ Unlike the “bits” of conventional computers, each qubit can exist in a superposition of two states at the same time, enabling quantum computers to process information in a radically different way, sometimes much faster than a classical computer.

In order for a quantum computer to be realized, it is necessary to build structures that can support qubits, keep them stable, and manipulate them. Qubits can be realized using photons, trapped ions/atoms, or electrons. It has been suggested that it would be advantageous to develop quantum computing systems based on silicon hardware,^38^ to help with interfacing quantum computers and their classical counterparts. There are many challenges for the implementation of silicon-based quantum computers, some of which relate to the fabrication of the devices. It is conceivable that DNA nanotechnology could play a role here, providing a way to make nanoscale structures that could not be synthesized with standard top-down methods.

**Figure 3:**
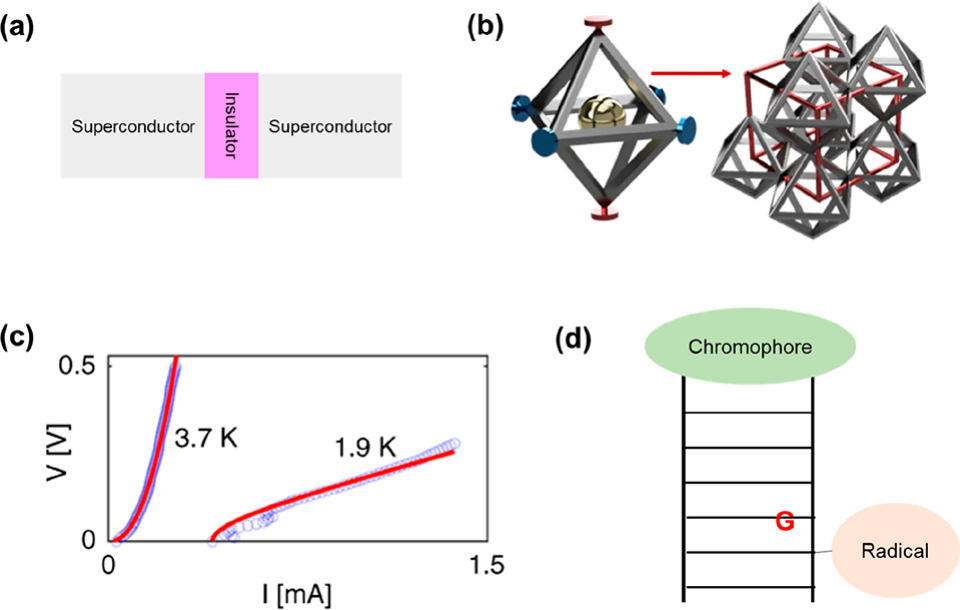
(a) Schematic illustration of a Josephson junction, consisting of a thin layer of insulating material sandwiched between two pieces of superconductor. (b) Assembly of DNA octahedra into a superlattice that was then coated with silica and niobium. There is a nanoparticle inside each octahedron. (c) Current–voltage characteristics of the resultant superlattice at temperatures of 3.7 K (just below the superconducting transition temperature) and 1.9 K. The data for 1.9 K has been fitted with the *I*–*V* characteristic of a Josephson junction. (b and c) From ref (40). CC BY 4.0. (d) Schematic diagram of the DNA structures described in ref (41), where the chromophore (hole donor) covalently links the two DNA strands. As indicated, only one guanine residue is present and a radical is attached to the DNA. The resultant structure can be used to generate a three-spin system.

Some quantum information processing systems make use of Josephson junctions (Figure 3a), consisting of two regions of superconducting material separated by a small insulating region across which electrons can tunnel. A Josephson junction is well-suited to the creation of qubits, and a variety of circuit designs can be used.^39^ Interestingly, DNA nanostructures can be used to assemble three-dimensional arrays of Josephson junctions^40^ (Figure 3b). The process began with the assembly of octahedra, in which each edge consisted of a six helix DNA origami bundle. The octahedra were assembled into a lattice before being coated with silica and niobium. In the resultant structure, superconductivity began at 3.8 K. Further characterization suggested that the lattice comprised a three-dimensional array of Josephson junctions (Figure 3c), such a structure being unattainable with conventional methods. This indicates how DNA nanotechnology could in future potentially help to address challenges involved in fabrication of quantum computing hardware, overcoming limitations of conventional nanofabrication methods. However, a great deal of work remains. Not only will it be necessary to demonstrate that DNA-templated structures can support stable qubits, but massively scaled-up production will be required.

Qubits can also be realized using organic chromophores and acceptor molecules. When the chromophore is photoexcited and transfers charge to the acceptor, this sometimes results in the creation of a spin qubit pair. The chromophore and acceptor can be held in position relative to each other with the use of a DNA scaffold, and the addition of a covalently bound radical could enable development of a three-spin system^41^ (Figure 3d). It has been noted that the use of chemistry and molecular engineering for quantum information systems is potentially a very powerful approach.^42^ Considering this and the other possibilities mentioned above, there already appears to be a reason to believe that DNA nanotechnology methods could have an impact in the area of quantum computing, despite the fact the latter field is still in its infancy. It may be helpful to combine DNA-scaffolded quantum information systems with DNA-templated nanophotonic structures (Nanophotonics).

### Nanophotonics

DNA nanotechnology has a plethora of applications in photonics,^8,43,44^ relying on nanoscale patterning and precise spatial arrangement of cargoes. Here we focus on selected examples.

**Figure 4:**
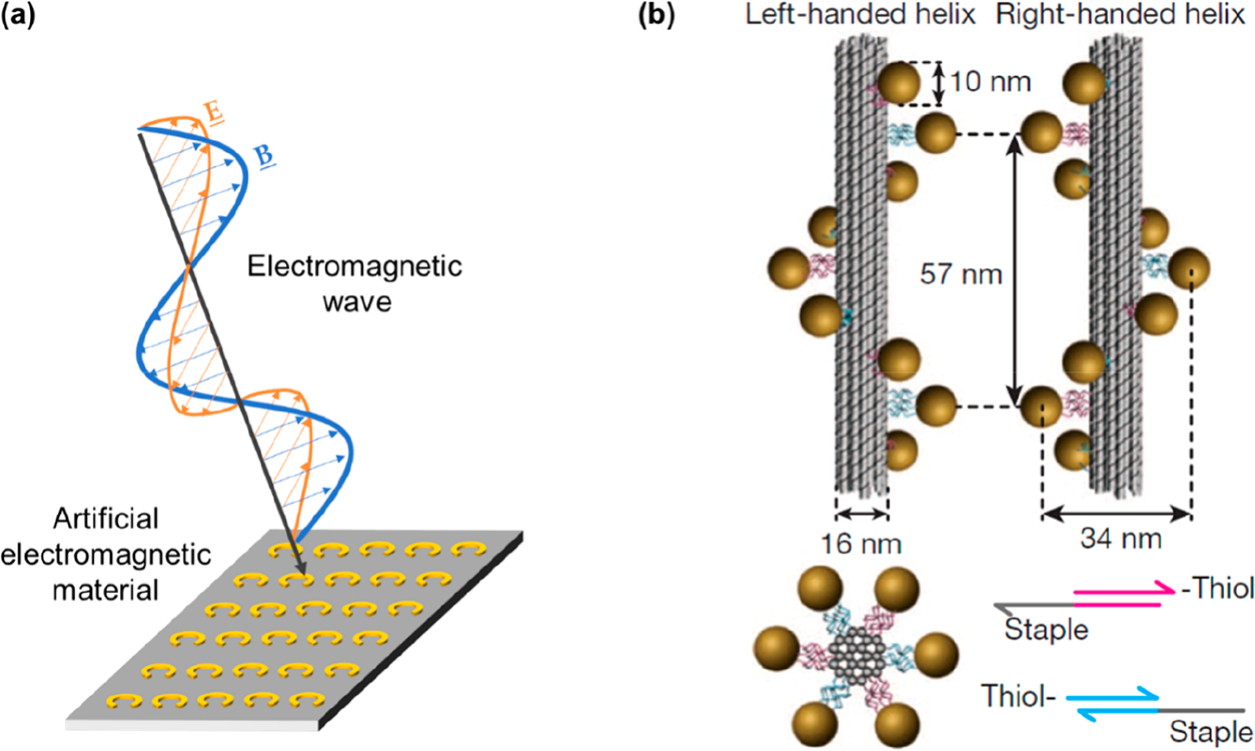
(a) Depiction of an artificial electromagnetic material (otherwise known as an optical metamaterial). The periodicity and feature size are significantly smaller than the wavelength of the electromagnetic wave, which interacts with the metamaterial as if it is an effective medium with engineered electromagnetic properties. (b) Gold nanoparticles arranged on a DNA origami bundle via thiol-modified linkers. Reprinted with permission from ref (52). Copyright 2012 Springer Nature. The two designs have opposite chirality, and this affects the way in which the structures interact with circularly polarized light, such that the circular dichroism spectrum of one exhibits a flipped sign relative to that of the other.

The notion of artificial electromagnetic materials (AEMs) was conceived over a century ago;^45^ fabricating arrays of conducting objects within a nonconducting matrix can result in a composite material that achieves bespoke electromagnetic properties. The vital prerequisite for the macroscopic composite is that it possesses a periodic structure with active features of dimensions and lattice spacing smaller than the length of the electromagnetic wave (Figure 4a). Indeed, if the size of the active features is sufficiently small, then these may act as an effective medium; i.e., the electromagnetic wave will experience the material as a monolithic entity. Examples of AEMs (also known as optical metamaterials) include, among other examples, materials with negative refractive indices and photonic crystals.^46^ The 1940s–1970s saw the accelerated development of AEMs in the microwave region (wavelengths of 30–0.1 cm), but until recently AEMs in the optical regime (400–700 nm), also known as optical metamaterials, were unmanufacturable. However, such materials are of considerable interest as they open the door to new ways of manipulating light, providing functions such as enhanced imaging capability or invisibility cloaks.

To produce a macroscale AEM will require hundreds of billions of active features, manufactured and assembled with exceptionally high fidelity and precision. The utilization of DNA nanotechnology for the fabrication of nanophotonic devices offers a number of compelling advantages, which have been demonstrated over the past decade in a number of studies, two of which amply illustrate its power: nanocavities and chiral structures.

Nanocavities are used to confine light using resonating modes at subwavelength scales. These have seen much use in quantum optical studies, in particular the creation of hybrid systems with nanocavities and single emitters (fluorophores or quantum dots), as shown for example in ref (47). The fabrication of such systems demands the deterministic placement of the emitters within the nanocavity, a task requiring accuracy orders of magnitude smaller than the wavelength.^48^ Gopinath et al.^49^ pioneered the use of DNA structures to control the position of a dye molecule within a photonic crystal cavity (PCC); by targeting the dye to different locations on a DNA structure located within the PCC, they demonstrated tunable emission corresponding to the electric field intensity within the PCC. The same group has gone on to develop control over the relative angle between the dipole of fluorescent dye and the polarization of the incident light, thereby governing device brightness.^50^

DNA nanotechnology has also opened up avenues in the study of chiral structures. In its optical sense, chirality allows a structure to differently absorb left- and right-handed circularly polarized light. Once more DNA nanotechnology’s ability to self-assemble these structures has propelled the emergence of a significant body of study using such systems,^51^ catalyzed by the marker laid down by Kuzyk et al.^52^ This study used a DNA nanorod as a scaffold to attach a helical string of gold nanospheres with a designed chiroptical response (Figure 4b). Left- and right-handed arrangements of the nanosphere were both shown to generate the characteristic bisignate circular dichroism spectra, centered at the resonant frequency of the individual nanosphere.

Overall, DNA nanotechnology has become a go-to solution for basic research in nanophotonics, but as yet there are very few commercialization successes to celebrate. Further technical development is required to de-risk the transition away from conventional materials, particularly in the context of scaling up to larger areas and mass production. Detailed economic assessment and life cycle analysis would be of particular benefit.

### Biobatteries

**Figure 5:**
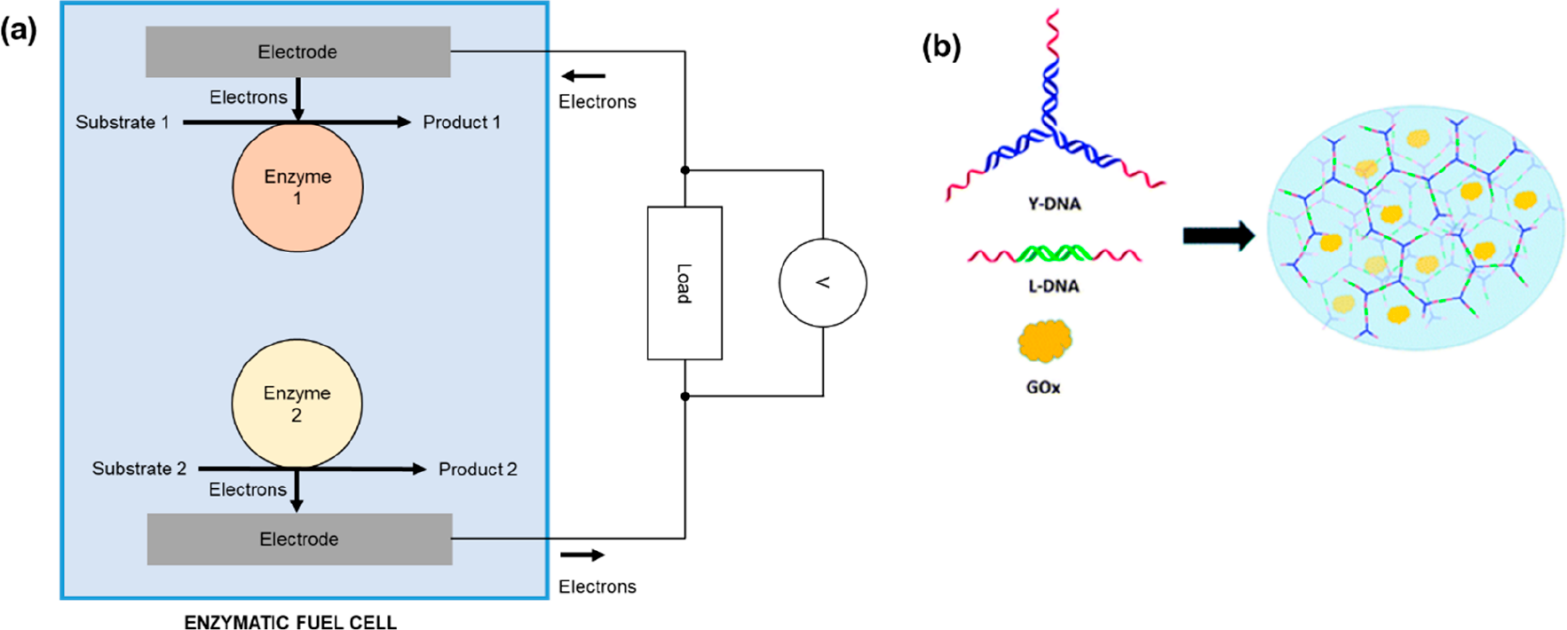
(a) Schematic illustration of the mechanism of an enzymatic fuel cell. The enzymes may be immobilized on the electrodes (perhaps covalently attached or trapped with polymers) or floating freely in solution. Additional redox-active compounds (not shown) may be added as mediators to enable electron transfer to the electrodes. In some implementations a semipermeable membrane (not shown) may be present in the cell to separate the two electrodes. The reaction catalyzed by the enzymes pushes/pulls electrons into/out of the electrodes, and this drives current flow through an external resistive load, across which a voltage may be measured as shown. (b) Encapsulation of enzyme GOx (glucose oxidase, yellow splotch) in a DNA hydrogel made from L-DNA linkers and Y-DNA three-way junctions. The DNA linkers and Y-shapes shown on the left assemble into the hydrogel shown on the right, in which GOx molecules are trapped. Reproduced with permission from ref (56). Copyright 2015 Royal Society of Chemistry.

Climate change and increasing use of electrical devices are driving research on new technologies for the energy sector. Bioengineering has the potential to make a valuable contribution to these efforts, and the term “electrosynbionics” has been coined to describe the “creation of engineered devices that use components derived from or inspired by biology” for electricity generation, use, and storage.^53^ This includes biophotovoltaics and biobatteries, among other technologies. Enzymatic fuel cells are a type of biological battery in which reactions catalyzed by enzymes generate a flow of electrons (Figure 5a). In one particularly interesting example, 13 different enzymes were used and a maximum current of 6 mA cm^–2^ was achieved.^54^ It was suggested that this device could have an energy storage density (in terms of energy stored per kilogram) an order of magnitude higher than lithium ion batteries.

DNA nanotechnology has a potential role to play in the development of the next generation of enzymatic fuel cells. As will be described shortly, a DNA-based hydrogel can be used as a medium for an enzymatic fuel cell, where a hydrogel consists of a network of linked polymers that contains a significant amount of absorbed water. Various DNA hydrogels have been reported, including one that was described as a “mechanical metamaterial”.^55^ Another technique for hydrogel formation involves structures called “Y-DNA” and “linkers”, and this was the approach used for the DNA hydrogel biobattery^56^ (Figure 5b). The Y-DNA was a three-way junction made from double-stranded DNA segments, with single-stranded sticky ends at all three termini. The linkers were duplexes with single-stranded overhangs at both ends. The enzyme glucose oxidase and mediator Fc-COOH were added to the mixture of DNA components. The resulting gel was applied to a stainless steel mesh anode and used with an air-breathing cathode, giving rise to an enzymatic fuel cell. Upon fuel addition, the maximum power density was approximately 300 μW cm^–2^, over 6.5 times the value observed in the absence of enzyme. It was later demonstrated that redox mediators could bind to the DNA, potentially providing a way to enhance electron transfer to the electrode.^57^ On the basis of recent news stories from the researchers and funders, further work appears to be in progress.^58−60^

DNA hydrogels share with origami structures the potential for straightforward assembly under benign conditions, and in both cases the product poses a minimal hazard, unlike more conventional devices that rely on more dangerous materials. For future development of DNA hydrogel biobatteries, it will be important to maximize energy and power density by perfecting the electron transfer pathway and choice of enzymes/substrates. The longevity and stability of the battery will need to be optimized, and the end-of-life disposal route must be confirmed.

## Discussion and Conclusion

Here, we have discussed examples of the use of DNA nanotechnology for applications in photonics and electronics. In addition to the case studies presented, it is worth noting that DNA nanostructures can be used in combination with other lithography techniques^61^ such as nanosphere lithography^62,63^ or top-down methods.^24,64−66^ In all the examples we considered, DNA nanotechnology offers great advantages in spatial precision and versatility while enabling assembly under benign conditions. Despite this, these approaches have not yet been fully exploited and further development is required for the full potential to be realized, particularly in connection with scaling up to commercial production levels. One aspect of this is the preparation of the DNA itself.

For many applications, chemical synthesis of oligonucleotides would be prohibitively expensive. DNA synthesis costs continue to fall, but alternative approaches are also being explored, for example using “biotechnological mass production”.^67^ Widespread deployment of new DNA synthesis methods could make DNA nanotechnology solutions more cost-effective, as has been demonstrated in the biomedical arena by modeling the economics of DNA nanostructure-based drug delivery.^68^

A second aspect of scaling up is the fidelity of assembly of nanostructures into bigger structures or arrays with a large surface area, as recently reviewed in ref (69). Several research groups have made impressive advances in this area, including surface-assisted assembly^70^ of tessellating origami triangles over 18.75 cm^2^ and the realization of supershapes using criss-cross assembly of origami slats.^71^

In general, translation of DNA nanotechnology research would be facilitated by a more problem-driven approach, where the design of devices is shaped by a detailed understanding of the needs of a particular target market and a focused device specification. Coupled with effective means to reduce cost and to scale up, this attitude has the potential to enable DNA nanotechnology to underpin a new generation of exciting products for photonic and electronic applications.
